# Detection of Human Endogenous Retrovirus K (HERV-K) Transcripts in Human Prostate Cancer Cell Lines

**DOI:** 10.3389/fonc.2013.00180

**Published:** 2013-07-09

**Authors:** Lorenzo Agoni, Chandan Guha, Jack Lenz

**Affiliations:** ^1^Department of Pathology, Albert Einstein College of Medicine, Bronx, NY, USA; ^2^Department of Radiation Oncology, Albert Einstein College of Medicine, Bronx, NY, USA; ^3^Department of Genetics, Albert Einstein College of Medicine, Bronx, NY, USA

**Keywords:** endogenous retroviruses, HERV-K, prostate cancer, cancer, unconventional splicing, RT-PCR, evolution

## Abstract

Human endogenous retroviruses (HERVs) are transcribed in many cancers including prostate cancer. Human endogenous retrovirus K (HERV-K) of the HML2 subtype is the most recently integrated and most intact retrovirus in the human genome, with many of the viral genomes encoding full- or partial-length viral proteins. To assess transcripts of HERV-K in prostate cancer cell lines and identify the specific HERV-K elements in the human genome that are transcribed, reverse transcriptase-PCR (RT-PCR) and cDNA sequencing were undertaken. Strand-specific RT-PCR, plasmid subcloning, and cDNA sequencing detected the presence of HERV-K(HML2) coding strand transcripts within four prostate cell lines (LNCaP, DU145, PC3, and VCaP). RT-PCR across splice junctions revealed splicing variants for *env* gene mRNA in three cell lines, two involving previously undescribed alternative splice sites. To determine the HERV-K loci from which the transcripts arose, RepeatMasker was used to compile a list of over 200 HERV-K internal genome segment fragments and over 1,000 HERV-K solo long terminal repeat (LTR) fragments in the human genome. Surprisingly, the sequences identified from internal positions of the viral genome were mostly smaller segments, while the LTRs were relatively intact. Possible reasons for this are discussed. The transcripts in the cell lines tested, arose from several HERV-K loci, with some proviruses being detected in multiple cell lines and others in only one of the four used. In some instances, transcripts from viral antisense strands was also detected. In addition, transcripts from both strands of solo LTRs were detected. These data show that transcripts from HERV-K loci commonly occur in prostate cancer cell lines and that transcription of either strand can occur. They also emphasize the importance of single nucleotide level analysis to identify the specific, individual HERV-K loci that are transcribed, and indicate that HERV-K expression in prostate cancer warrants further study.

## Introduction

Human Endogenous Retroviruses (HERVs) exist as the integrated form of retrovirus DNA, called proviruses, at many loci in the human genome. They are the result of ancient retroviral infections of the human germline cells during evolution (1). There are many different species of endogenous retroviruses in the humans today, and collectively they comprise about 8% of the human genome. Since the divergence of the human and chimpanzee lineages approximately six million years ago, the only retrovirus known to have entered the genome of the human lineage is the HML2 subset of human endogenous retrovirus K (HERV-K) (2–8). Since it is the newest HERV, HERV-K(HML2) thus has had the least time to accumulate mutations and constitutes the most intact set of retrovirus in the human genome (2, 9–16). Some HERV-K(HML2) proviruses entered the germline of the human lineage earlier in evolutionary time in the common ancestors of humans and other catarrhines (2, 17–19). In the absence of selection pressure on the host to maintain the proviral genomes in an intact form, recombination events, and various other types of mutations inevitably accumulate in the proviruses over evolutionary time. The most common mutation of HERV-K is the formation of solo long terminal repeats (LTRs), which occurs by homologous recombination between the two LTRs of an individual provirus (16, 20). For HERV-K, solo LTRs outnumber proviruses by more than 10 to 1 (16). No HERV-K proviral locus has been found that produces fully functional, infectious virions, although the components to assemble an infectious virus genome exist in the human genome today (14). There are over 1,000 HERV-K(HML2)-containing loci in the human genome today, that range from solo LTRs and fragments of proviruses to almost intact, full-length proviruses. Many of the HERV-K proviruses are sufficiently intact to encode functional proteins (2, 12–16), and these proteins have been suggested to contribute to diseases including cancers (21–25, 39).

Human endogenous retrovirus K is transcribed to various extents in several human diseases including cancer (26–30), HIV infection (31–35), and autoimmune disorders (36, 37). Human-specific HERV-K(HML2) elements in the human genome are approximately 99% identical in pairwise comparisons to each other, and many of the differences among them are single base pair changes. Thus the techniques used to detect HERV-K transcription often do not allow a determination of which specific viral loci in the human genome were the source of the detected RNAs. Individual loci differ in the integrity of specific open reading frames (ORFs), functionality of the encoded proteins, and the capacity of DNA sequences flanking the viral DNA as well as unique mutations within individual loci to affect transcription. In certain instances, transcription of specific HERV-K loci has been recognized (38–41), although for most HERV-K loci, particularly solo LTRs, HERV-K transcription has not been assessed. In addition, many HERV-K loci are situated within introns of genes, often in the reverse transcriptional orientation, and they may be transcribed as a segment of the gene being expressed (42). Transcription of multiple HERV types including HERV-K was reported in human prostate cancer (PrCa) and occurs to varying extents (43–46). To understand the biological role, if any, and potentially to predict functionality of HERV-K in human diseases, a thorough analysis of viral transcription that includes solo LTRs, viral fragments, DNA strand, and genomic loci of origin is necessary. As a step toward these ultimate goals, we analyzed four PrCa cell lines by strand-specific reverse transcriptase-PCR (RT-PCR). Sequencing of PCR products was used to determine the loci of specific transcripts.

## Results

### HERV-K loci in human genome

To define the loci of origin for individual HERV-K transcripts, it was first necessary to generate a comprehensive list of the HERV-K segments in the human genome. This was accomplished using the Table Browser tool (47) at UCSC Genome Browser (48) and the GRCh37/hg19 human genome assembly. The sequences and genomic positions of LTR and internal genome segments were downloaded using RepeatMasker (49) and RepBase definitions (50) for LTR5_Hs, LTR5A, or LTR5B for the HERV-K LTRs, and HERVK-int for the internal HERV-K segments.

**Figure 1:**
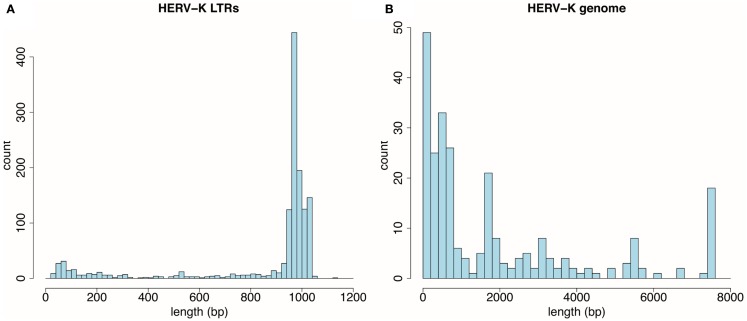
**Distribution by size of HERV-K components detected by RepeatMasker in the UCSC Genome Browser, GRCh37/hg19 human genome assembly**. **(A)** Thousand three hundred and sixty-one HERV-K LTR fragments are plotted as function of 20 bp length windows. **(B)** Two hundred fifty-five HERV-K provirus internal genome segments are plotted as a function of 200 bp length windows.

Thousand three hundred and sixty-one LTRs and 255 HERV-K internal genome fragments were obtained. This is somewhat larger than the number of loci assessed by Subramanian et al. (16) mainly because RepeatMasker identified multiple fragments derived from individual HERV-K genomes, and these were counted separately here. RepeatMasker identified the fragments separately when HERV-K loci were disrupted by deletions, insertions, or extensive substitution mutations. In Figure 1, each locus was plotted as a function of its length. The LTRs were mostly intact and not broken into fragments. The peak around 970 bp (Figure 1A) reflected the size of the LTRs of the most recently acquired, *Homo sapiens*-specific proviruses. In contrast, the viral internal genome segments were mostly disrupted into smaller fragments (Figure 1B). Only 26 loci had full-length or near full-length internal sequences. HERV-K proviruses were categorized as type I or II (51) depending on the presence of a 292-bp stretch spanning the *pol-env* gene boundary (type II) or the deletion of that segment (type I). The 26 loci included 18 that fell into the 7,400- to 7,600-bp window, plus 8 type I proviruses where RepeatMasker separately identified the 5′ part of the internal sequences in the 5,400- to 5,600-bp window and the 3′ part in the 1,600- to 1,800-bp window. These loci and a few additional loci relevant to this study are listed in Table 1.

**Table 1:** **Full-length HERV-K (HML-2) proviruses and their locations in the human genome^a^**.

Provirus	Type	Length	NCBI accession #	Chrom.	UCSC coordinates^a^
K101	I	9,175	AF164609	22q11.21	18,926,187–18,935,361
K102	I	9,180	AF164610	1q22	155,596,457–155,605,636
K103^b^	I	968	AF164611^b^	10p12.1	27,182,399–27,183,366
K104	II	9,446	AF164612	5p13.3	30,486,760–30,496,205
K105^c^		968	AF165235–AF165234^d^	Un_g1000219	175,210–176,178
	I	6,361	and JQ790992		
K106	I	9,159	AF165240–AF165241^d^	3q13.2	112,743,124–112,752,282
K107/K10	I	9,180	AF164613	5q33.3	156,084,717–156,093,896
K108	II	17,975^e^	AF164614	7p22.1	4,622,057–4,640,031
K109	II	9,422	AF164615	6q14.1	78,426,662–78,436,083
K110	I	9,232	AF165258–AF165259^d^	1q23.3	160,660,575–160,669,806
K111	I	9,160	GU476554	11q23.3	118,591,724–118,600,883
K112/K33	II	9,463	DQ112100	10p14	6,866,141–6,875,603
K113^f^			AF387847–AF387849^d^	19p12	21,841,536–21,841,541^f^
	II	9,473	and AY037928		
K114/K36	II	9,466	DQ112101	11q22.1	101,565,794–101,575,259
K115			AF387846–AF387848^d^	8p23.1	7,355,397–7,364,859
	II	9,463	and AY037929		
K116/K50B	I	9,180	DQ112147	3q27.2	85,280,336–185,289,515
K117/K41	II	9,457	DQ112140	12q14.1	58,721,242–58,730,698
K118/K(II)	I	9,123	AB047240.1	3q12.3	101,410,737–101,419,859
K(I)	II	9,138	AB047209	3q21.2	125,609,302–125,618,439
K4	I	6,373	DQ112097	1p31.1	75,842,771–75,849,143
K5	I	7,229	DQ112093	4q32.3	165,916,840–165,924,068
K50F	II	9,518	DQ112151	19q13.12	37,597,549–37,607,066
K51	I	6,738	DQ112098	19p12	22,757,824–22,764,561
K60	I	9,657	DQ112108	21q21.1	19,935,621–19,940,996
3p12.3	II	8,685^g^	ND^h^	3p12.3	75,600,465–75,609,150
4p16.1	II	9,594	ND^h^	4p16.1	9,659,580–9,669,174
K17B	II	9,584	DQ112124	4p16.1	9,123,515–9,133,075
4p16.3	II	9,588	ND^h^	4p16.3	3,979,051–3,988,639
K7	II	9,554	DQ112131	11p15.4	3,468,656–3,478,209
8p23.1c	II	9,527	ND^h^	8p23.1	12,073,970–12,083,497
K27	II	10,117	DQ112118	8p23.1	8,054,700–8,064,221
KO1D130352	II	9,516	DQ112134	8p23.1	12,316,492–12,326,007

*^a^Locations in the human genome according to the UCSC Human Genome Assembly, February 2009 (GRCh37/hg19)*.

*^b^K103 is present as a solo LTR in the genome assembly. The accession number is for the full-length provirus*.

*^c^K105 is present as a solo LTR (chrUn_gl000219:175,110–176,278) inserted in centromeric region (CER satellite) DNA*.

*^d^Accession numbers for the flanking sequences*.

*^e^K108 is present as a tandemly duplicated provirus in the genome assembly*.

*^f^K113 is absent from the genome assembly (GRCh37/hg19); coordinates for the duplicated target sequence at insertion site are annotated*.

*^g^Most of the 5′ LTR is deleted*.

*^h^These proviruses are present in the UCSC Human Genome Assembly, February 2009 (GRCh37/hg19), but have not been characterized independently and do not have accession numbers*.

Similarly, the genomic coordinates for all known genes were obtained using the knownGene table function in the UCSC Genes track for whole genes, exons, introns, 5′ untranslated regions (5′ UTRs), 3′ untranslated regions (3′ UTRs), and coding sequences (CDSs) (Table 2). Thirty-three percent of LTRs and 31% of internal HERV-K genome sequences were located within transcribed portions of UCSC genes genes, which is similar to the fraction of the human genome encompassed by the transcribed portions of genes. Four percent of the viral loci overlap with exons, mainly with untranslated regions (UTRs). Three of the viral loci overlapped CDSs of putative human genes. None of those three genes have been firmly characterized, and for two of them, the overlapping segments were short. Details of the overlaps are described below in the Section Materials and Methods.

**Table 2:** **Location of HERV-K loci relative to human protein-coding genes and their components**.

repName byRepeatMasker(total number)	Gene features						
	Intergenic^a^ (%) +intragenic (%)	Codingcapacity	Gene (same:opposite)orientation	Exon (same:opposite)orientation	5′ UTR (same:opposite)orientation	3′ UTR (same:opposite)orientation	CDS (same:opposite)orientation
LTR5_Hs, LTR5A, LTR5B (1,361)	906 (67%) + 455 (33%)	Coding	381 (99:284)^b^	16 (8:9)^b^	5 (3:2)	11 (5:6)	2 (0:2)
		Non-coding	87 (53:36)^b^	20 (17:4)^b^	–	–	–
HERVK-int (255)	175 (69%) + 80(31%)	Coding	56 (14:42)	5 (3:2)	2 (1:1)	2 (1:1)	1 (0:1)
		Non-coding	24 (14:11)^b^	10 (9:2)^b^	–	–	–

*^a^Intergenic is defined as outside the transcribed portions of genes, and intragenic is defined as spanning the transcribed portion of a gene*.

*^b^A number of intersections were counted for both directions when a locus had two genes in transcribed in opposite directions*.

### HERV-K transcription in prostate cancer cell lines

**Figure 2:**
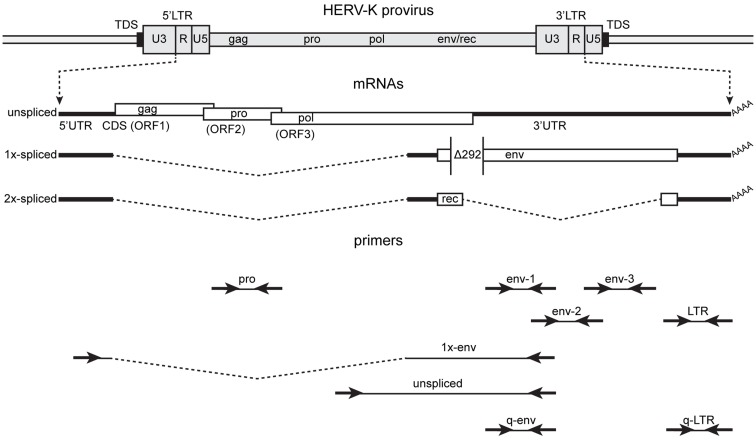
**Structure of the HERV-K genome and spliced mRNAs showing the primers used for reverse transcription and PCR**. A genetic map of a HERV-K provirus (darker gray) inserted into host genome (lighter gray) is shown with target duplicated sequence (TDS) indicated as black boxes. The unspliced primary viral transcript, 1×-spliced *env* mRNA and 2×-spliced *rec* mRNAs are shown below the viral genome with 3′ poly(A) tails indicated (AAAA). The *env* mRNA shows the position of the 292-nt deletion (Δ292) of type I HERV-K proviruses that spans the *pol-env* junction. Type II HERV-K provirus transcripts contain these nucleotides. Positions of the PCR primer pairs are shown at the bottom with the names by which they are identified throughout the paper. The dashed, angled line shows the excised intronic sequences that the 1×-env primer pair was designed to cross.

**Figure 3:**
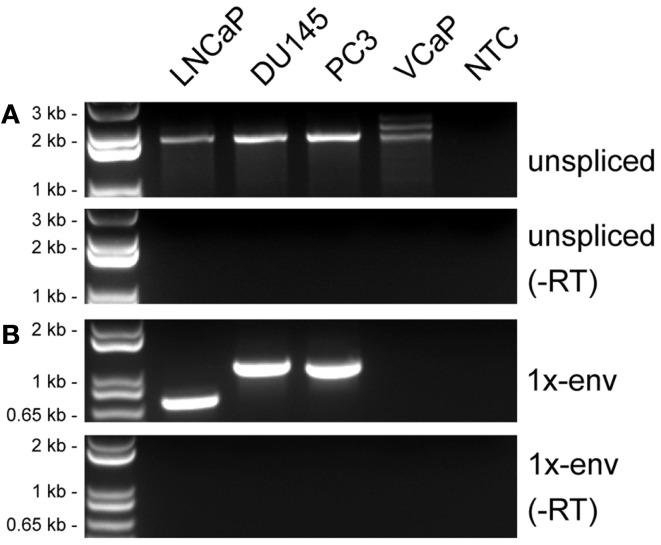
**Detection of HERV-K transcripts in four prostate cancer cell lines**. RT-PCR was performed to detect viral sense strand transcripts specifically, and the products were resolved by electrophoresis. **(A)** RT-PCR products from a primer pair designed to detect unspliced viral RNAs (unspliced). **(B)** RT-PCR products from primers designed to detect RNAs spliced at the conventional *env* mRNA splice junction (1×-env). Genomic positions of the primers are shown in Figure 2. Parallel controls were performed without reverse transcriptase (−RT) and with no added template (NTC, no template control) as controls to exclude DNA contamination. DNA size markers are shown on the left.

The general approach used to analyze HERV-K RNA expression was to perform RT-PCR on RNAs isolated from prostate cancer cell lines (Figure 2). These analyses were undertaken as part of ongoing studies of these tumors. Primers were designed in conserved stretches of viral sequences and were expected to amplify most of the HERV-K loci in the human genome identified by RepeatMasker above. Nonetheless, it is possible that particular loci were not recognized by the particular primer pairs used. Transcripts were amplified using RNAs isolated from four PrCa cell lines. RT-PCR using a primer pair to detect a 2-kb amplicon spanning the *pol-env* junction in unspliced viral RNA (“unspliced,” Figure 2) is shown in Figure 3A. Two kilobase products were detected in all four PrCa cell lines. Parallel amplification reactions were performed without RT and were uniformly negative, thus showing that amplification was from RNA templates and not from any potentially contaminating genomic DNA (Figure 3A). The larger band in VCaP cells corresponded to that from type II proviruses, while the smaller band observed in all four lines corresponded to type I proviruses.

### Multiple HERV-K loci are active

Individual proviruses inevitably accumulate unique mutations over evolutionary time. These differences and the sequences flanking the proviruses could affect the expression of specific HERV-K loci. They can also be used to identify the specific viral loci from which transcripts originated. Pairwise comparisons of the sequences of human-specific, HERV-K proviruses showed that they were about 99% identical. Thus sequencing of the PCR products was necessary to identify the specific loci of origin for the transcripts, and it is was important for the sequence reads to be long enough to encompass the limited polymorphisms among the newest proviruses in the human genome. Sequence reads were matched to the closest HERV-K locus in the human genome. Many sequence reads matched a genome provirus 100%. Some had one or more mismatches, but all of these matched a genomic locus by>99%. The differences may be due either to polymorphisms among the HERV-K loci in the human population, the extent of which is largely unknown, or to misincorporation errors during RT-PCR. Both RT and the PCR polymerase can cause nucleotide misincorporations.

To determine whether or not the HERV-K transcripts detected in PrCa cells arose from a single HERV-K locus or multiple loci in the human genome, the PCR products shown in Figure 3A were sequenced (Table 3). Direct sequencing of the amplicons from DU145 and LNCaP cells allowed identification of the transcripts as containing mutations unique to HERV-K102 and HERV-K118 proviruses, respectively, thus showing that they derived principally from these loci (Table 3) (hereafter proviruses are called simply K102, etc.). PC3 and VCaP showed mixed peaks in the chromatographs, and thus the PCR products were shotgun cloned into plasmid vectors, and at least seven individual clones of each were sequenced. Both lines contained transcripts from K118, while K102 was observed in PC3 and K50F was found in VCaP. Based on the nearly equal mixed peak heights in the chromatographs of the initial PCR products (data not shown), it is plausible to estimate that the proportion of transcription of the two loci was roughly comparable. In summary, the sequencing analysis showed that the transcripts originated from at least three different HERV-K loci in the human genome, and that they partially varied among the cell lines. The analysis detected only the most abundant cDNAs that were amplified, and more extensive sequencing might identify additional transcribed loci of lower abundance.

**Table 3:** **Cell lines where transcripts from specific HERV-K proviruses were detected**.

Genomic locus	PCR fragments (amplicons)	
	Unspliced	1×-env^a^
K118 (3q12.3)	LNCaP^b^	LNCaP
	PC3^c^	
	VCaP^c^	
K102 (1q22)	DU145^b^	–
	PC3^c^	
K50F (19q13.12)	VCaP^c^	–
K108 (7p22.1)	–	DU145
K60 (21q21.1)	–	PC3

*^a^From direct sequencing of PCR products in Figure 3B*.

*^b^From direct sequencing of PCR products in Figure 3A*.

*^c^From sequencing of plasmid subclones of PCR products in Figure 3A*.

### Some HERV-K transcripts are spliced in prostate cancer cell lines

In addition to assessing which proviruses had sufficient integrity for the RNAs to be spliced, the detection of viral splicing would provide further evidence that the RT-PCR products derived from viral RNAs and not from any potentially contaminating DNA. To investigate whether the HERV-K transcripts could be spliced, we performed strand-specific RT-PCR across one of the viral splicing junctions in the four PrCa cell line. Using primers designed to detect the 1×-spliced *env* mRNA (Figure 2), three out of four cell lines were found to be positive (Figure 3B), although the sizes of the products were different in the different lines. To investigate the basis for this, the products were sequenced (Table 3). This analysis showed that a different locus was responsible for the predominant spliced cDNA product in each line, K108 in DU145, K118 in LNCaP, and K60 in PC3.

**Figure 4:**
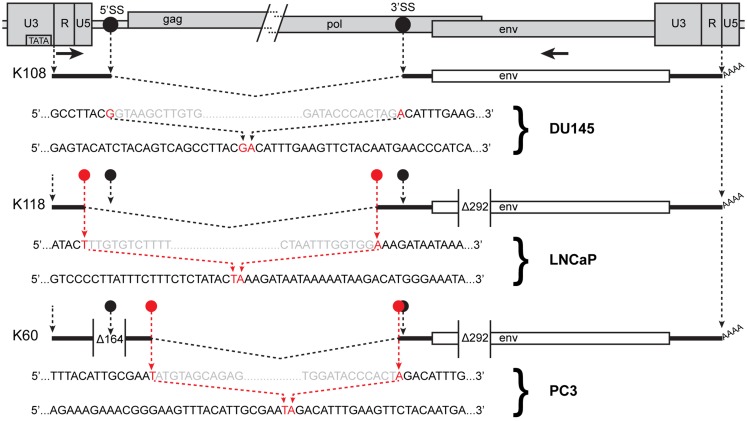
**Human endogenous retrovirus K RNA splice sites determined by sequencing of RT-PCR products**. The 5′ and 3′ portions of a HERV-K provirus are shown at the top separated by / and /. 5′SS and 3′SS indicate the conventional splicing sites of HERV-K, and their positions are marked as black circles. The positions of the outer PCR primers used for the nested PCR are shown as arrows. Structures of the *env* spliced RT-PCR products from the cell lines and proviruses indicated are diagrammed below the viral genome. Dashed angled lines show the excised introns. Red circles show the positions of the sequences where unconventional splicing occurred. For each spliced product, the top sequence shows the inferred primary transcript sequence determined from that of the cognate genomic locus, and the bottom sequence shows the sequence of the RT-PCR product. Red nucleotides were joined in the spliced product. Gray nucleotides show the ends of the excised sequences. The positions of the 292-nt deletion definitive of type I proviruses and a 164-nt deletion in the K60 provirus relative to other HERV-K proviruses are shown.

K108 is one of the most intact HERV-K proviruses in the human genome, having full-length ORFs for all viral proteins (2, 12). Splicing of RNA from this provirus in DU145 cells occurred at the canonical *env* mRNA splice sites in the viral genome (Figure 4). Detection of the expected splice junction for K108 *env* mRNA further confirmed that the RT-PCR products were derived from RNA templates. Splicing for both K118 and K60 was detected at unique, non-canonical sites, and the positions were different for each (Figure 4). For K60 in PC3 cells, the 5′ splice site matched the consensus sequence for the minor spliceosome (52) and was located 435 nt downstream of the canonical 5′ splice site. The 3′ splicing site matched no apparent consensus sequence and was only 6 nt downstream of the canonical *env* mRNA 3′ splicing site. The alternative splice sites used and the presence or absence of the 292-bp *pol-env* junction characteristic of type I HERV-K proviruses (Figure 4) accounted for the differences in sizes of the RT-PCR products in Figure 3B. The absence of unspliced products corresponding to K108 and K60 (Table 3) may be due to unspliced transcripts from these proviruses being less abundant than those from the other proviruses in these cells and/or transcripts from these proviruses being more completely spliced.

The spliced HERV-K products from DU145, LNCaP, and PC3 cells were not generated in the absence of RT (Figure 3B), showing that they were indeed derived from RNA templates. The possibility that they were derived from spliced pseudogenes was also considered by searching existing human genome sequences for the sequences that we detected using BLAT. No such products were detected. Moreover the splicing of K108 RNA exactly at the canonical sites as expected provided a control that expected splicing was detectable under the conditions used. Thus it appears that the unexpected use of non-canonical splice sites for some HERV-K transcripts accurately reflected unusual splicing that occurred in the PrCa cell lines used.

### Solo LTRs are actively transcribed

The question whether solo LTRs are transcribed was also addressed. As these, like viral LTRs in general, contain the viral U3 region transcriptional regulatory elements, they thus have the potential to initiate transcription (53, 54). In addition, many HERV-K solo LTRs are present within transcription units of cellular genes and may be transcribed as segments of such units. Since solo LTRs outnumber proviruses containing the viral internal sequences (16), we hypothesized that the levels of their RNAs might be higher than those from HERV-K internal sequences. If, alternatively, LTR transcripts were predominantly components of longer viral transcripts originating from full-length HERV-K proviruses, then the levels would be similar.

**Figure 5:**
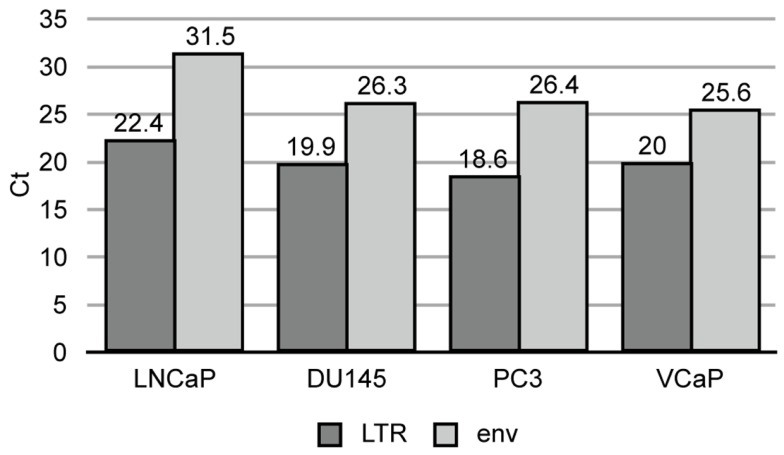
**Quantitative RT-PCR of HERV-K LTR and *env* sequences in prostate cancer cell lines**. Threshold cycle is shown for the viral components in each cell line.

To answer this question, two-step, quantitative, RT-PCR was performed with one primer pair designed in the LTR and one in the *env* gene (q-LTR and q-env, Figure 2). The q-LTR pair was positioned in the R portion of the LTR, and the q-env pair was positioned so that it detected both unspliced and 1×-spliced *env* mRNA. In all four PrCa cell lines, the average difference of threshold cycle (Ct) between transcription levels of LTRs and *env* was about 7.2 Ct (Figure 5), which corresponds to roughly 100-fold or greater levels of LTR RNA than unspliced or 1×-spliced RNA. Since the unspliced and 1×-spliced RNAs have two copies of the LTR R region for each copy of *env* (Figure 2), the great excess of LTR transcripts presumably reflects a large excess of transcripts derived from solo LTRs.

### HERV-K elements are transcribed from both strands

**Figure 6:**
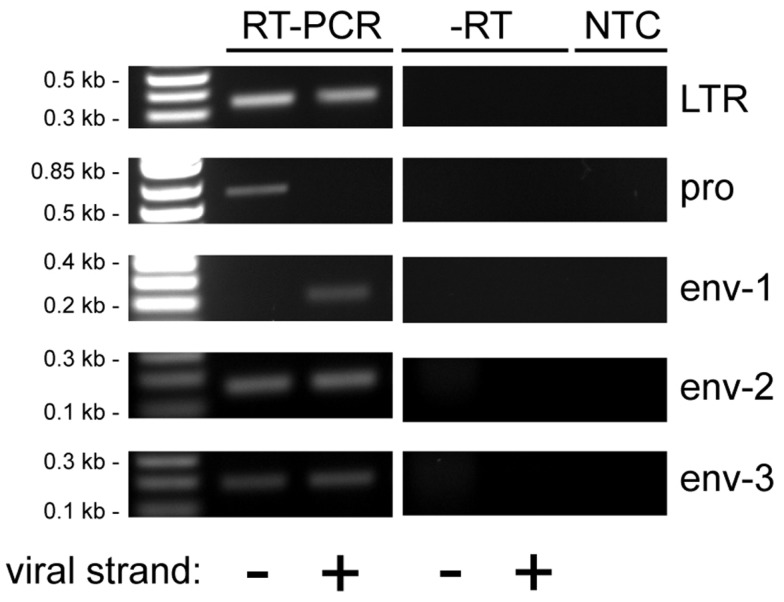
**Reverse transcriptase-PCR analysis of HERV-K transcripts in LNCaP cells**. Strand-specific, RT-PCR was performed on RNA isolated from LNCaP cells for five different positions in the HERV-K genome using the primer pairs identified in Figure 2, and the products were resolved by electrophoresis. Viral strand indicates RT-PCR products that originated from viral sense (+) or antisense (−) RNAs. Parallel controls were performed without reverse transcriptase (−RT) and without adding any RNA template (NTC, no template control) to exclude DNA contamination. DNA size markers are shown on the left.

Human endogenous retrovirus K LTRs, like those of other retroviruses, contain the viral enhancers and promoters that may potentially drive transcription of the viral sense strand (53, 54). They also contain signals for polyadenylation (55). In addition, proviruses and solo LTRs may be integrated into other transcription units and be transcribed as part of them (42). Thus transcribed HERV-K sequences may arise from either the sense strand, the antisense strand, or both, depending on where active promoters are located. Viral sequence-containing transcripts may include introns in pre-mRNAs, long non-coding RNAs, standard viral transcripts, solo-LTR initiated RNAs, LTR-polyadenylated transcripts, and even short RNAs of unknown significance. To assess transcripts in the PrCa cell line more comprehensively, five different primer pairs for short PCR amplicons were designed for LTR, *pro*, and *env* genes (Figure 2). For the *env* gene, three non-overlapping regions were used. Sequencing was used to identify the loci of origin for the RNAs detected. By including either the forward or the reverse primer separately during the reverse transcription step, strand specificity of HERV-K transcripts could be determined. RT-PCR was performed on LNCaP cells (Figure 6). The primary PCR products were directly sequenced, and, in addition, the products were cloned into plasmid vectors, and 7–10 clones were sequenced for each PCR product (Table 4).

**Table 4:** **Human endogenous retrovirus K proviruses detected in LNCaP cells by sequencing plasmid subclones**.

Genomic locus	Viral strand	PCR fragments (amplicons)^a^				
		LTR	pro	env-1	env-2	env-3
K(I) (3q21.2)	Rev	1/8	2/9	6/8	–^b^	5/7^c^
	Fwd	–	–	–	–	–
K106 (3q13.2)	Rev	–	1/9	–	3/9^c^	1/7
	Fwd	–	–	–	–	–
K50F (19q13.12)	Rev	–	–	2/8	5/9	–
	Fwd	–	–	–	–	–
Fragment 11q12.3	Rev	–	–	–	1/9	1/7
	Fwd	–	–	–	–	–
K118 (3q12.3)	Rev	–	–	–	–	–
	Fwd	–	–	10/10^c^	10/10	7/7
Fragment 7q34	Rev	–	6/9	–	–	–
	Fwd	–	–	–	–	–
Solo LTRs (multiple loci)	Rev	7^d^/8^c^	–	–	–	–
	Fwd	10/10^c^	–	–	–	–

*^a^Numerators are the number of clones detected from the individual locus indicated, and denominators are the number of clones sequenced. Fwd and Rev indicate whether the RNA matched the forward (sense) or reverse (antisense) strand of the viral DNA genome*.

*^b^–Indicates not detected*.

*^c^Ambiguous amplicon*.

*^d^In this sample 2 sequences may belong to LTRs of full-length HERV-Ks or to solo LTRs but the sequence is ambiguous*.

Analysis of sense strand transcripts from the internal portion of the HERV-K genome in LNCaP cells showed that 27/27 clones derived from HERV-K118 (Table 4), similar to what was observed above for the unspliced primer pair spanning the *pol-env* junction (Table 3). In contrast, sequencing of products the HERV-K antisense strand showed that they were derived from at least five different loci, including full length, recently inserted, HERV-Ks and more ancient HERV-K loci. Among these, the older HERV-K(I) locus was the most frequently detected one during the sequencing of plasmid clones that were derived from HERV-K antisense transcripts (39%) (Table 4). Other proviruses from which antisense transcripts were detected were K106, K50F, and two unnamed, fragmented loci (Table 4). Two of the proviruses identified by this approach, K50F and the fragment at 11q12.3, are known to be inserted in introns of human genes and in the opposite transcriptional orientation. Thus the viral antisense strands for these might be a consequence of being within the primary transcripts of the host genes. The other four HERV-K loci are not within introns of human genes, and the basis for transcription of their antisense strands is unclear.

Reverse transcriptase-PCR products from the LTR region were also analyzed (Table 4). Both strands of the HERV-K LTR were represented. For 94% (17/18) of the clones sequenced, the best genomic locus or loci matching the obtained sequences were not in the LTRs of proviruses with internal viral sequences. Instead one or more solo LTRs were the loci of origin. Since the primer pair was in a well-conserved component (the R region) of LTR, it was not always possible to assign a sequence uniquely to a single solo LTR locus. Nonetheless, these data provided further evidence proving that solo LTR transcription is widespread, in addition to showing that transcripts of both the sense or antisense strands of various HERV-K proviruses and solo LTRs were transcribed.

## Discussion

Human endogenous retrovirus K(HML2) transcripts were detected in all four prostate cancer cell lines examined. These included transcripts of both viral strands and transcripts that were spliced. HERV-K transcripts were previously detected in prostate cancer and include loci in addition to those identified here (35, 45). The results here employed cDNA sequencing to distinguish from which among the highly similar HERV-K loci in the human genome the most predominant transcripts arose. Certain HERV-K loci in the human genome, K118 and K102, were transcribed in multiple prostate cancer cell lines, raising the possibility that the genomic insertion sites, mutations unique to them, and/or epigenetic states of these proviruses make them prone to transcription in these cells lines. Other HERV-K loci were detected only in individual lines. However, detection here was limited by the number of plasmid clones sequenced, and deeper sequencing might reveal transcripts from more HERV-K loci. Nonetheless the loci identified here likely reflected the most abundantly transcribed loci in these cell lines. This study emphasizes the importance of analyzing HERV-K transcripts at the single nucleotide level to distinguish the specific loci from which they originated.

An additional finding here was that HERV-K solo LTRs were abundantly transcribed in these cell lines. This is not surprising in that these are more abundant than HERV-K internal segments in the human genome (16). However, their abundance complicates identification of the specific, individual loci of origin, and their detection raises questions about the mechanisms that cause them to be transcribed. Many are present in introns of human coding genes (Table 2), and one possibility is that they may be transcribed as components of the primary transcripts of these genes.

One unexpected finding here was the less intact nature of HERV-K internal genome segments relative to LTRs as detected by RepeatMasker (Figure 1). The LTR sizes clustered around large, full-length, and near full-length segments, while those of internal genome segments clustered at the very low end (Figures 1A vs 1B). Multiple factors could contribute to the internal segments being recognized as separate fragments by RepeatMasker. One is that if a portion of a repeated element suffered sufficiently extensive mutation over evolutionary time that RepeatMasker no longer recognized that portion as part of the element, then the two flanking segments of the element would be reported as different fragments. Likewise, if genetic breakage and rejoining events including insertions, deletions, or other rearrangements occurred, then an element would be reported by RepeatMasker as separate segments. The internal segment of HERV-K is longer than the LTR, roughly 7,500 vs. 1,000 bp, respectively, and thus would be more likely to have suffered mutations. The 1,361 LTR fragments encompassed 1,162,766 bp. The mean length of 864 bp corresponded to about 86% of a full-length LTR or roughly 1.2 fragments per LTR. The 255 HERV-K internal segment fragments encompassed 487,441 bp. The mean length of 1,911 bp corresponded to about 25% of a full-length internal segment or roughly 4 fragments per internal genome segment. The difference between LTRs and internal segments is greater than could be caused even if half the internal HERV-K segments were type I proviruses (missing the 292-bp) and thus recognized as two fragments. There are many potential explanations for this unexpected difference. One is that RepeatMasker may detect some HERV-K internal fragments that include some evolutionarily older and thus more fragmented, HERV-K variants than it does with LTR segments. Another is gene conversion. HERV-K LTRs have been well-documented to undergo gene conversion events among paralogous LTR loci (4, 18, 56). The greater abundance of LTRs might have caused them to undergo more inter-locus gene conversion events resulting in more homogenous sequences and thus being recognized as intact by RepeatMasker compared to internal segments. Other unprecedented and seemingly less plausible alternatives might include different mutation rates or effects on host fitness. The genetic basis is for the unexpected difference between LTRs and internal segments requires further study and might turn out to be biologically interesting.

Another unexpected finding was the detection of splicing at non-standard sites. Use of the expected splice sites was observed for K108 which is a type II provirus and one of the most intact loci in the human genome, containing full-length ORFs for all viral proteins (2, 12). The non-standard splicing involved type I proviruses (K118 and K60). The 292-nt that are deleted in type I HERV-K proviruses span a segment from just downstream of the standard, *env* 3′-splice site and encompass the 5′-splice site for the second intron of HERV-K (Figure 4). Perhaps this deletion and/or other mutations in these proviruses affect splice site usage.

These studies bear on long term goals of understanding HERV-K expression in cancer cells, and possibly exploiting it for T-cell based immunotherapy in combination with conventional chemo-RT therapy. The detection of HERV-K transcripts in prostate cancer cell lines adds to the list of human tumors in which RNAs from this retrovirus have been observed and raises the point that further study of the virus in this disease is warranted, as it is in other diseases. Immune responses against HERV antigens have been reported in human diseases (27, 28, 33, 57–59), and such responses might be of significance for certain types of human cancers if HERV-K is expressed. Detailed analyses such as those performed here provide a step toward clarifying the spectrum of HERV-K expression in human tumor cells.

## Materials and Methods

### Determination of HERV-K locus number and size in human genome

The Table Browser tool (47) at UCSC Genome Browser (62) website^1^ was used to download the sequences and genomic positions of HERV-K elements in the human genome sequence. RepeatMasker (49) definitions based on RepBase (50) were filtered by repName matching for LTR5_Hs, LTR5A, or LTR5B for the HERV-K LTRs and HERVK-int for proviral internal HERV-K genome segments. R software (60)^2^ was used to plot the loci against their size for LTRs (Figure 1A) and HERVK-int (Figure 1B).

Similarly, the genomic coordinates for all known genes in human genome were obtained using knownGene in UCSC Genes track, separately for whole gene, exons, introns, 5′ UTRs, 3′ UTRs, and CDSs. The HERV-K and gene coordinate data were uploaded on Galaxy (61)^3^ for further analysis. To determine the overlap of each locus with annotated genes in the human genome, we used the Intersect tool (version 1.0.0) to compare genomic positions of HERV-K loci with known genes and their subcomponents (exons, introns, 5′ UTRs, 3′ UTRs, and CDSs). Intersection of at least 1 nt was considered as overlapping to generate the data summarized in Table 2.

The finding that three HERV-K fragments overlapped with CDSs was unexpected and was analyzed further. In one instance, a 772-bp fragment of a solo LTR was found to be located in the TBC1D29 gene on chromosome 17. The LTR fragment was in the opposite orientation as the gene, and the 3′ end of the fragment comprised the last 19 coding nucleotides of the ORF, two tandem stop codons, and the small 3′ UTR of one isoform of TBC1D29. This gene is a novel, putative TBC-1 domain protein, which are a family of Rab-GAP GTPase activator proteins.

A second instance was only a short 49 bp LTR_5B segment in the opposite orientation as gene R3HCC1 on chromosome 8. The LTR segment formed the 3′ part of the second coding exon of the gene, through the third nucleotide before the splice junction. Multiz alignments on the UCSC Genome Browser showed that a similar sequence was in the rhesus macaque (*Macaca mulatta*) genome assembly, and no comparable element has been found for mouse and dog, consistent with a HERV-K insertion after the catarrhine divergence from other primates. However, similar sequences also have been identified for elephant and opossum. In particular, the elephant sequence encoded an identical amino acid sequence except at one position, which is not consistent with a HERV-K element. Given the short extent of this sequence and the apparent existence of orthologous sequences in elephant and opossum, it is difficult to discern if this is a bona fide HERV-K segment or an unusual sequence convergence, and caution should be reserved.

The third was a viral internal segment that overlapped a cDNA encoding a single exon transcript AX747630 on chromosome 17 with an ORF encoding 180 amino acids. The N-terminal 98 amino acids of the HERV-K Gag protein provided the first portion of the putative human protein. The same HERV-K insertion was present in the rhesus macaque genome assembly, and the CDSs of the human and macaque ORFs were 84% identical. Whether a real protein is produced from the ORF, and whether or not it has any function are unknown.

### Cell lines and culture conditions

Four PrCa cell lines (LNCaP, DU145, PC3, and VCaP) were purchased from ATCC (Manassas, VA, USA). Cell lines were grown in 5% CO_2_ in a humidified 37 °C incubator to 90% confluence.

### RNA extraction

Total RNA was extracted using Trizol (Invitrogen). RNA was then subjected to DNAse-I digestion (TURBO DNA-free kit – Applied Biosystems #AM1907) to remove any genomic DNA contamination. RNA quality and concentration were determined by evaluation of rRNA bands in agarose gel electrophoresis and by NanoDrop spectrophotometric analysis (Thermo Scientific).

### RT-PCR and sequencing

To identify the specific active HERV-K loci, 1 μg of each RNA was used for gene-specific RT-PCR. Primers were designed in well-conserved segments of HERV-K genome to ensure amplification of as many loci as possible. A set of primers was designed across the viral *env* splicing junction to identify the singly spliced variant of HERV-K transcript. Parallel controls were performed to detect beta-actin and GAPDH transcripts. Primer sequences were as follows:

unspliced-Fwd: 5′-CTGGTGCATGGAAGATTGGT-3′
unspliced-Rev: 5′-CACCGCACTATTGGCCACA-3′
1×-env(1)-Fwd: 5′-AGGGAAAAACCGCCTTAGGG-3′
1×-env(1)-Rev: 5′-CACCGCACTATTGGCCACA-3′
1×-env(2)-Fwd: 5′-TGCGGGCAGCAATACTGCT-3′
1×-env(2)-Rev: 5′-CGCACTATTGGCCACACATTC-3′
LTR-Fwd: 5′-CGTGGGAAGGGAAAGACCTGA-3′
LTR-Rev: 5′-AGCAGACAAACATGTGAACAAAGG-3′
pro-Fwd: 5′-CCCCACAAAACCCCCACAG-3′
pro-Rev: 5′-GATTTCCGCACCCCATTGTTG-3′
env-1-Fwd: 5′-CATGGTAAGCGGGATGTCACT-3′
env-1-Rev: 5′-CACCGCACTATTGGCCACA-3′
env-2-Fwd: 5′-GTGTGGCCAATAGTGCGGT-3′
env-2-Rev: 5′-ATTCCTTTTTCTCCCCATTCCCAG-3′
env-3-Fwd: 5′-GCTGGTGAGAGCAAGAGAGG-3′
env-3-Rev: 5′-CAATGCAACTCCTGCTACAGC-3′
q-env-Fwd: 5′-TCACATGGTAAGCGGGATGTC-3′
q-env-Rev: 5′-CGCACTATTGGCCACACATTC-3′
q-LTR-Fwd: 5′-AGGGAAAAACCGCCTTAGGG-3′
q-LTR-Rev: 5′-AGCAGACAAACATGTGAACAAAGG-3′
hACTB-Fwd: 5′-CTGGCACCACACCTTCTACA-3′
hACTB-Rev: 5′-GCTGGGGTGTTGAAGGTCT-3′
hGAPDH-Fwd: 5′-AGATCATCAGCAATGCCTCCT-3′
hGAPDH-Rev: 5′-AGTCTTCTGGGTGGCAGTG-3′

The reverse transcription reactions (SuperScript III First-Strand Synthesis System – Invitrogen #18080-051) were performed using 1 μg RNA in an initial volume of 10 μL with 1 μL of dNTP mix 10 mM, gene-specific primer for a final concentration of 2 μM and DEPC-treated water. After an initial denaturation step, 5 min at 65°C followed by 1 min at 4°C, 2 μL of 10× RT buffer, 4 μL of 25 mM MgCl_2_, 2 μL of 0.1 M DTT, 40 U RNaseOUT, and 200 U SuperScript III RT enzyme were added to reach a final volume of 20 μL. Gene-specific primers used were unspliced-Rev and 1×-env(1)-Rev for experiments shown in Figure 3; LTR-Rev, pro-1-Rev, env-1-Rev, env-2-Rev, and env-3-Rev for experiments to detect viral plus strand transcripts in Figure 6; and LTR-Fwd, pro-1-Fwd, env-1-Fwd, env-2-Fwd, and env-3-Fwd for experiments to detect viral minus strand transcripts in Figure 6. The RT elongation step was performed at 50°C for 50 min, followed by enzyme heat inactivation at 85°C for 5 min. After brief cooling of the sample at 4°C, digestion of residual RNA was performed with RNAse-H 2 U at 37°C for 20 min. Parallel experiments in which no RT enzyme was added were simultaneously carried out.

PCR was performed with 2 μL of RT product, 200 nM primers 200, Platinum PCR SuperMix (Invitrogen), and nuclease-free water to a total volume of 20 μL. After a denaturation step at 94°C for 2 min, 30 cycles of denaturation-annealing-elongation were performed, followed by final elongation at 72°C for 5 min. Denaturation was performed at 94°C for 25 s, annealing for 25 s, and extension at 72°C for each experiment as follows. Detection of unspliced amplicon (Figure 2A) was performed with annealing temperature of 58°C followed by extension time of 2.5 min. Detection of 1×-env amplicon (Figure 2B) was performed with a reaction at annealing temperature of 58°C followed by extension time of 3 min, 2 μL of a 1:200 dilution of the PCR product was then used in nested PCR reaction with the 1×-env(2) primers, and annealing temperature of 61°C followed by extension time of 3 min. Detection of LTR, pro, env-1, env-2, and env-3 amplicons (Figure 6) was performed with annealing temperature of 55°C followed by extension time of 1 min.

Electrophoresis of PCR products was performed in 1% agarose gels, PCR products obtained were purified (PCR purification – Qiagen), and recovered cDNA was sequenced. PCR products were cloned (TOPO TA cloning – Invitrogen Inc.) into pCR4 plasmids using MACH-1 competent cells and sequenced.

### Identification of HERV-K loci

Each sequence obtained, trimmed of any vector and primers sequences, was aligned to the human genome using BLAT software (62). The sequence was assigned to the locus obtaining the highest identity score (Tables 3 and 4). If more than one locus had the same identity score, the sequence was considered ambiguous (Table 4). All sequences meeting the minimum length requirement of GenBank (>200 bp) were submitted there (accession numbers KF254334–KF254392).

### Quantitative RT-PCR

To quantify HERV-K transcripts, 1 μg of RNA from each set was used for two-step, quantitative RT-PCR (qRT-PCR) (Superscript III First-Strand – Invitrogen) and PowerSybr Green (AppliedBiosystems). RT (SuperScript III First-Strand Synthesis System – Invitrogen #18080-051) of 1 μg RNA was performed in an initial volume of 10 μL with 1 μL of 10 mM dNTP mix, 1 μL of 50 μM Oligo(dT)_20_ primer, and DEPC-treated water. After an initial denaturation step of 5 min at 65°C followed by 1 min at 4°C, 2 μL of 10× RT buffer, 4 μL of 25 mM MgCl_2_, 2 μL of 0.1 M DTT, 40 U RNaseOUT, and 200 U SuperScript III RT enzyme were added to reach a final volume of 20 μL. The RT elongation step was performed at 50°C for 50 min, followed by enzyme heat inactivation at 85°C for 5 min. After brief cooling of the sample at 4°C, digestion of residual RNA was performed with 2 U RNAse-H at 37°C for 20 min. Parallel experiments with no RT enzyme were carried out simultaneously.

One microlitter of a 1:10 dilution of the RT product was then added to 2× SYBR Green PCR Master Mix (AppliedBiosystems #4367659) and nuclease-free water to a final volume of 8 μL. Primers q-env-Fwd and q-env-Rev were designed in a well-conserved region of HERV-K genome common to unspliced and once-spliced transcript variants downstream of the Δ292 deletion characteristic of type I HERV-Ks. Primers q-LTR-Fwd and q-LTR-Rev were designed in a well-conserved region of HERV-K LTR corresponding to the R region. Two housekeeping genes, human beta-Actin (hACTB) and human GAPDH (hGAPDH), were used as endogenous controls for normalization. Three replicates per sample were used in 384-well plate PCR system (7900HT – AppliedBiosystems) with cycling conditions of 50°C for 2 min, 95°C for 10 min, 40 cycles of 95°C for 10 s, 60°C for 20 s and 72°C for 30 s. Data analysis was performed with SDS 2.4 software (AppliedBiosystems).

## Conflict of Interest Statement

The authors declare that the research was conducted in the absence of any commercial or financial relationships that could be construed as a potential conflict of interest.

## Footnotes

^1^http://genome.ucsc.edu/

^2^http://www.R-project.org

^3^http://galaxyproject.org/
